# Prolonged Myelosuppression due to Progressive Bone Marrow Fibrosis in a Patient with Acute Promyelocytic Leukemia

**DOI:** 10.1155/2019/1616237

**Published:** 2019-11-27

**Authors:** Yuta Inagawa, Yukiko Komeno, Satoshi Saito, Yuji Maenohara, Tetsuro Yamagishi, Hiroyuki Kawashima, Taku Saito, Keiko Abe, Kuniko Iihara, Yasumasa Hatada, Tomiko Ryu

**Affiliations:** ^1^Department of Hematology, Japan Community Healthcare Organization (JCHO) Tokyo Yamate Medical Center, Tokyo, Japan; ^2^Department of Internal Medicine, JCHO Tokyo Yamate Medical Center, Tokyo, Japan; ^3^Department of Gastroenterology, JCHO Tokyo Yamate Medical Center, Tokyo, Japan; ^4^Department of Orthopaedic Surgery, Faculty of Medicine, The University of Tokyo, Tokyo, Japan; ^5^Division of Orthopedic Surgery, Department of Regenerative and Transplant Medicine, Niigata University Graduate School of Medicine and Dental Sciences, Niigata, Japan; ^6^Department of Pathology, JCHO Tokyo Yamate Medical Center, Tokyo, Japan

## Abstract

A 34-year-old woman was diagnosed with acute promyelocytic leukemia. Chemotherapy was administered following the JALSG APL204 protocol. Induction therapy with all-trans retinoic acid resulted in complete remission on day 49. She developed coccygeal pain from day 18, which spread to the spine and cheekbones and lasted 5 weeks. She had similar bone pain on days 7–10 of the first consolidation therapy and on days 4–12 of the second consolidation therapy. Oral loxoprofen was prescribed for pain relief. On day 33 of the third consolidation, white blood cell and neutrophil counts were 320/*μ*L and 20/*μ*L, respectively. After she developed epigastralgia and hematemesis, she developed septic shock. Gastroendoscopy revealed markedly thickened folds and diffusely damaged mucosa with blood oozing. Computed tomography revealed thickened walls of the antrum and the pylorus. Despite emergency treatments, she died. Bacterial culture of the gastric fluid yielded *Enterobacter cloacae* and enterococci growth. Collectively, she was diagnosed with phlegmonous gastritis. Retrospective examination of serial bone marrow biopsy specimens demonstrated progressive bone marrow fibrosis, which may have caused prolonged myelosuppression. Thus, evaluation of bone marrow fibrosis by bone marrow biopsy after each treatment cycle might serve as a predictor of persistent myelosuppression induced by chemotherapy.

## 1. Introduction

Acute promyelocytic leukemia (APL) is a subtype of acute myelocytic leukemia (AML) with the most favorable outcome [1, 2]. APL prognosis has dramatically improved since the introduction of all-trans retinoic acid (ATRA), a molecular targeted therapy which induces differentiation of APL cells [3]. ATRA plus chemotherapy has now become the standard therapy. Recently, ATRA plus arsenic trioxide, another molecular targeted therapy, has also been administered with or without chemotherapy in both frontline and relapsed APL cases [2].

Bone marrow fibrosis (BMF) is a histopathological process characterized by an increased deposition of reticulin fibers, and in some cases collagen fibers, in the bone marrow [4]. A wide variety of malignant and nonmalignant disorders are associated with BMF. Nonmalignant conditions underlying BMF include endocrine disorders, autoimmune diseases, and infections [4]. Regarding malignant conditions, primary myelofibrosis and secondary myelofibrosis (arising from polycythemia vera or essential thrombocythemia) are classified as Philadelphia-negative myeloproliferative neoplasms (MPNs) which are often, but not always, accompanied by *JAK2*, *CALR*, or *MPL* mutations [1]. Acute megakaryoblastic leukemia is the most common subtype of AML associated with BMF [1]. However, there are several previous reports of APL patients who presented with severe BMF at diagnosis [5–7].

Herein, we present details of a patient with APL, who suffered from fatal phlegmonous gastritis during prolonged myelosuppression caused by progressive BMF.

## 2. Case Presentation

A 34-year-old Japanese woman visited our hospital with purpura in the lower extremities. Blood tests showed pancytopenia (white blood cells (WBC) 1340/*μ*L (blast 1.5%, promyelocyte 21.5%, and neutrophil (Neu) 7.0%), hemoglobin 8.4 g/dL, and platelets 2.8 × 10^4^/*μ*L) and disseminated intravascular coagulation (fibrinogen 67 mg/dL, fibrin degradation products 93 *μ*g/mL, and D-dimer 32.7 *μ*g/mL). She was emergently hospitalized. A bone marrow aspiration smear showed 1.8% blasts and 88.1% promyelocytes and myeloperoxidase strongly positive. Auer bodies and Faggot cells were also observed. The patient was diagnosed with acute promyelocytic leukemia (APL). Leukemic cells were CD13(+), CD33(+), CD34(−), CD117(+), and HLA-DR(−). Leukemic cell karyotype was 46,XX,der(15)t(15;17)(q22;q12), ider(17)(q10)t(15;17) [20/20]. *PML-RARA* fluorescence in situ hybridization (FISH) was positive in 98.0% of cells (fusion signal : 2/PML : 1/RARA : 1 = 2.0% and fusion signal : 3/PML : 1/RARA : 1 = 96.0%, where the PML probe binds to 15q24 and the RARA probe binds to 17q21). Quantitative polymerase chain reaction (qPCR) of *PML-RARA* fusion mRNA was 7.0 × 10^4^ copies/*μ*gRNA. Bone marrow biopsy demonstrated hypercellular marrow without an increase in argyrophilic fibers. Erythroid cells and megakaryocytes were severely decreased.

**Figure 1:**
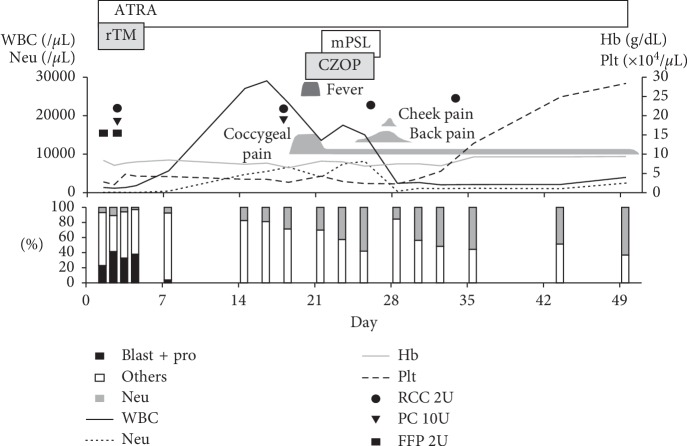
Clinical course of induction chemotherapy. On the horizontal axis, day 1 was set as the first day of induction chemotherapy. WBC, white blood cell; Neu, neutrophil; Hb, hemoglobin; Plt, platelet; Pro, promyelocyte; ATRA, all-trans retinoic acid; rTM, recombinant human thrombomodulin; mPSL, methylprednisolone; CZOP, cefozopran; RCC, red cell concentrate; PC, platelet concentrate; FFP, fresh frozen plasma.

Induction chemotherapy was performed with oral all-trans retinoic acid (ATRA) at a dosage of 45 mg/m^2^ as a single agent, following the JALSG APL204 protocol (Figure 1) [8]. On day 18, she experienced a sudden strong coccygeal pain while walking and became unable to walk. On day 20, she developed fever and cefozopran was administered. Since differentiation syndrome could not be ruled out, methylprednisolone 80 mg/day was administered for 4 days. Magnetic resonance imaging on day 21 demonstrated no obvious abnormality in the lower sacrum and coccyx. On day 24, she acquired back pain, which gradually worsened. On day 27, she had cheekbone pain, which disappeared the following day. Back pain disappeared on day 29, but coccygeal pain persisted. Oral loxoprofen was necessary for pain relief. On day 49, complete remission (CR) was confirmed by bone marrow examination and ATRA administration was discontinued. Bone marrow smear showed 0.6% blasts and 0.4% promyelocytes. Karyotype was normal. *PML-RARA* FISH was positive in 3.0% of cells (fusion signal : 2/PML : 1/RARA : 1 = 2.0% and fusion signal : 3/PML : 1/RARA : 1 = 1.0%). qPCR of *PML-RARA* fusion mRNA was 4.9 × 10^3^ copies/*μ*gRNA.

**Figure 2:**
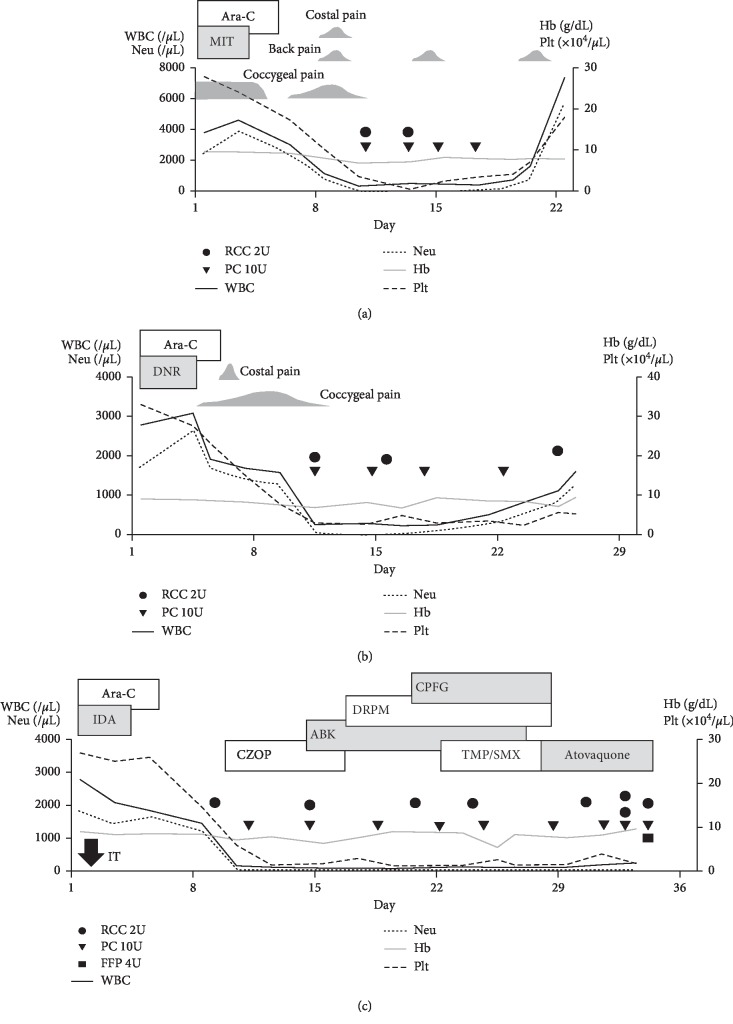
Prolonged myelosuppression during three courses of consolidation chemotherapy. (a) First course; (b) second course; (c) third course. As the consolidation therapy progressed, myelosuppression prolonged. Back pain and coccygeal pain became milder during the later course of consolidation therapy. On horizontal axis, day 1 was set as the first day of each course of chemotherapy. WBC, white blood cell; Neu, neutrophil; Hb, hemoglobin; Plt, platelet; RCC, red cell concentrate; PC, platelet concentrate; FFP, fresh frozen plasma; Ara-C, cytarabine; MIT, mitoxantrone; DNR, daunorubicin; IDA, idarubicin; CZOP, cefozopran; ABK, arbekacin; DRPM, doripenem; CPFG, caspofungin; TMP/SMX, trimethoprim/sulfamethoxazole; IT, intrathecal injection.

First consolidation chemotherapy involved cytarabine 200 mg/m^2^ for 5 days and mitoxantrone 7 mg/m^2^ for 3 days (Figure 2(a)). The patient had persistent coccygeal pain from the induction of chemotherapy until day 5. She experienced bone pain in the coccyx, spine, and costae on days 7–10. She again had back pain on days 14–15 and 18–19. Neutrophil count recovered on day 22 (WBC 7380/*μ*L, Neu 5680/*μ*L). Bone marrow aspiration/biopsy on day 35 demonstrated CR with 1.7% blasts and 0.5% promyelocytes. Karyotype was normal. *PML-RARA* FISH was negative. qPCR of *PML-RARA* fusion mRNA was undetectable.

Second consolidation chemotherapy involved cytarabine 200 mg/m^2^ for 5 days with daunorubicin 50 mg/m^2^ for 3 days (Figure 2(b)). The patient developed coccygeal bone pain on days 4–12 and costal pain on days 6–7. Neutrophil count recovered on day 25 (WBC 1600/*μ*L, Neu 1280/*μ*L). Bone marrow aspiration/biopsy on day 41 demonstrated CR with 1.2% blasts and 0.7% promyelocytes. Karyotype was normal. *PML-RARA* FISH was negative. qPCR of *PML-RARA* fusion mRNA was undetectable.

Third consolidation chemotherapy involved cytarabine 140 mg/m^2^ for 5 days with idarubicin 12 mg/m^2^ for 3 days (Figure 2(c)). On day 1, a peripherally inserted central venous catheter (PICC) was inserted. Intrathecal injection of methotrexate 15 mg, cytarabine 40 mg, and prednisolone 10 mg was also administered. Cytology of the cerebrospinal fluid was categorized as class I. To treat neutropenic fever, antibiotics were administered. Fever persisted and the PICC was removed. On day 25, the patient had bilateral pneumocystis pneumonia and trimethoprim-sulfamethoxazole was prescribed. Intravenous immunoglobulin was also administered. On day 28, she defervesced. Computed tomography on day 29 showed remission of ground-glass opacity. On day 30, due to abdominal discomfort, trimethoprim-sulfamethoxazole was replaced with atovaquone and other antibiotics were discontinued.

**Figure 3:**
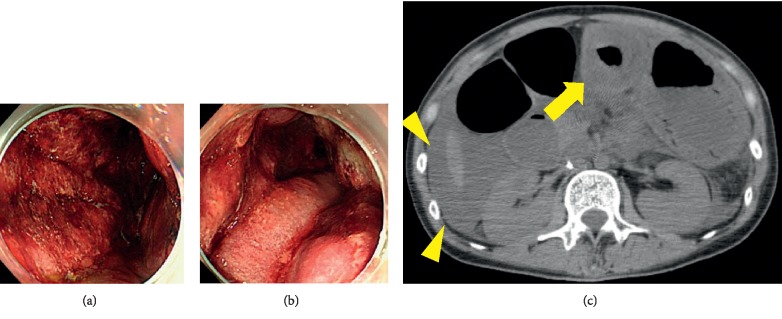
Phlegmonous gastritis. (a, b) Upper endoscopy. The gastric mucosa was reddish, and the folds were markedly thickened. (c) Abdominal CT. Fluid level was noted in the stomach. Arrow, thickened wall of the antrum and the pylorus with hypodense areas inside. Arrowheads, ascites.

On the night of day 32, the patient vomited twice. During the early morning on day 33, she presented with epigastralgia and hematemesis and then became drowsy. She was afebrile. Blood pressure and oxygen saturation were unmeasurable. She was diagnosed with septic shock. Her abdomen was soft and flat on palpation, with epigastric tenderness. A central venous catheter was inserted. Intravenous dopamine, blood transfusion, and fluid therapy were administered. Laboratory data showed mild elevation of C-reactive protein (CRP) (3.1 mg/dL) and liver dysfunction. Echocardiography demonstrated hypovolemic state with normal ejection fraction. Upper endoscopy (Figures 3(a) and 3(b)) revealed dark-red bloody fluid in the stomach. Gastric folds were markedly thickened. Gastric mucosa was diffusely damaged, and blood was oozing from all areas. No ulcers or pulsatile bleeding were confirmed. The esophagus and the duodenum appeared normal. Phlegmonous gastritis was suspected. She was transferred to an intensive care unit. For pain relief, pentazocine was infused. Norepinephrine was also administered. At midnight, she became unconscious. Cardiopulmonary resuscitation was performed. She was intubated and connected to a respirator. Her abdomen was distended. Computed tomography (Figure 3(c)) revealed markedly thickened wall of the antrum and the pylorus with hypodense areas. Massive ascites was also observed. Gasses and fluid in the intestines were compatible with paralytic ileus. When a nasogastric tube was inserted, relatively fresh bloody fluid came out, which was used for bacterial culture. Laboratory data showed multiorgan failure and elevated CRP (10.0 mg/dL). Cefozopran was started. Blood pressure went down despite blood transfusion. Cardiopulmonary arrest was confirmed. The approval for autopsy was not obtained from the family.

Bacterial culture of the gastric fluid yielded *Enterobacter cloacae*, *Enterococcus* species, and methicillin-resistant *Staphylococcus*. Together with the results of imaging studies, the patient was diagnosed with phlegmonous gastritis (PG).

**Figure 4:**
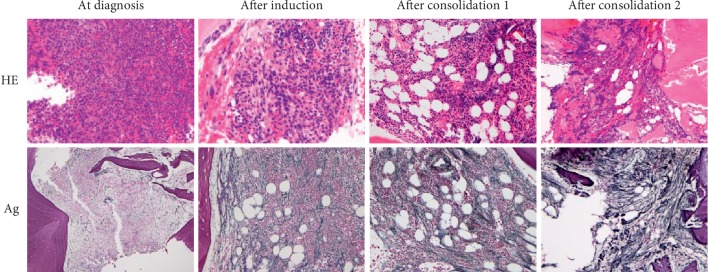
Serial bone marrow biopsies (hematoxylin-eosin (HE) and silver staining (Ag)). Only mild reticulin fibrosis was observed at the time of diagnosis. Fibrosis progressed as treatment progressed, and collagenous fibrosis was recognized. Magnification, 40x.

**Figure 5:**
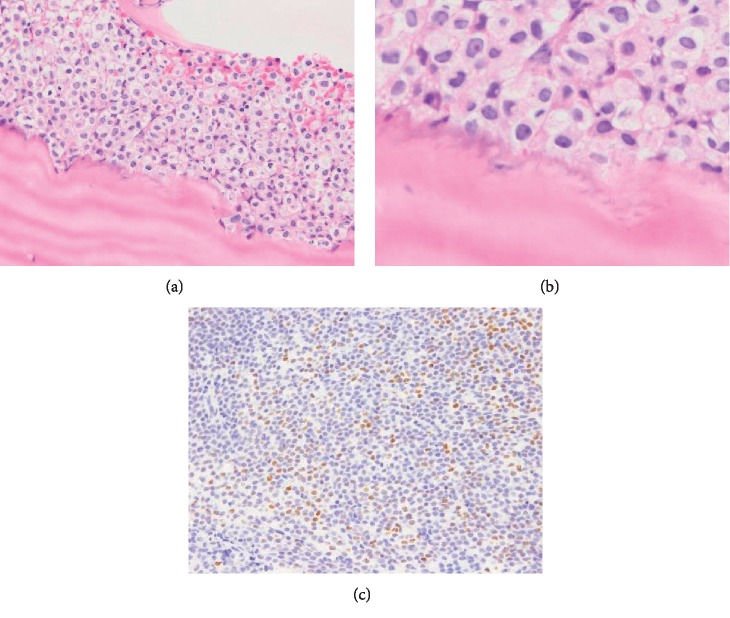
Histopathology of bone marrow biopsy at diagnosis. (a)-(b) Hematoxylin-Eosin stain showed that the bone trabecula border in contact with leukemic cells shows osteolysis-like appearance. (a) Magnification, 100x; (b) magnification, 400x; (c) parathyroid hormone-related protein stain. Positive cells were stained brown. Magnification, 100x.

**Figure 6:**
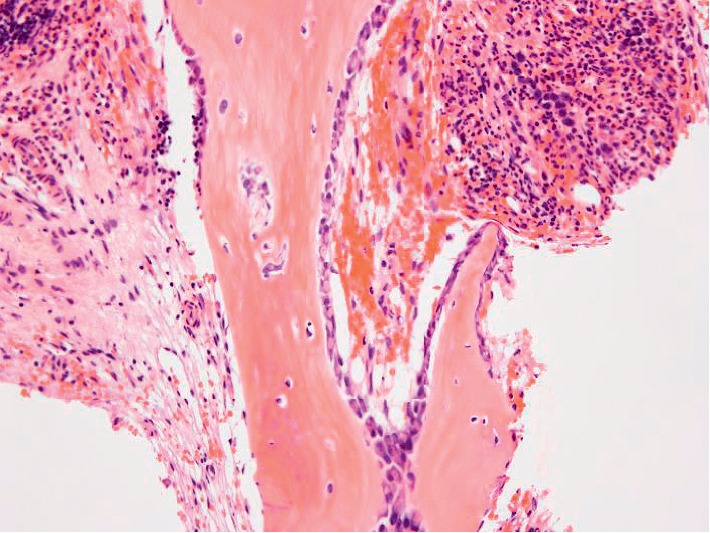
Multilayered osteoblasts in the bone marrow after induction chemotherapy. Osteoblasts multilayered along the trabecular bone. Magnification, 40x.

To investigate the cause of progressive myelosuppression, bone marrow biopsy samples were re-evaluated retrospectively (Figure 4). Each sample was classified as normocellular BM compatible with CR, and the number of megakaryocytes was within normal range. However, a silver stain demonstrated that BMF progressed rapidly after each cycle of consolidation chemotherapy, suggesting that prolonged myelosuppression was caused by progressive BMF. Argyrophilic fibers were increased. Intriguingly, at diagnosis, the bone trabecular border in contact with leukemic cells showed osteolysis-like appearance (Figures 5(a) and 5(b)). Immunostaining of PTH-related protein (PTH-rP) and receptor activator of nuclear factor kappa-Β ligand (RANKL), the molecules involved in bone remodeling, was performed. PTH-rP was positive in some of the leukemic cells (Figure 5(c)), while RANKL was negative (data not shown). Since these molecules activate osteoclasts, we performed TRAP staining to detect osteoclasts, which was negative (data not shown). In addition, immunostaining of osterix (Osx), an osteoblast-specific transcription factor, demonstrated multilayered osteoblasts along the trabecula in the bone marrow only after induction chemotherapy (Figure 6). Thus, prolonged myelosuppression was suspected to be caused by progressive BMF, although its exact mechanism was unclear.

## 3. Discussion

Bone marrow fibrosis (BMF) is characterized by an increased deposition of reticulin fibers, and in some cases collagen fibers, in the bone marrow [4]. BMF as a side effect of all-trans retinoic acid (ATRA) has been reported by Hatake et al. [9]. Eleven of thirteen (84.6%) APL patients who received ATRA as induction therapy developed collagenous fibrosis after 1–8 weeks. Fibrosis resolved spontaneously or after consolidation therapy. Five of sixteen (31.3%) APL patients who received chemotherapy alone, without ATRA, developed collagenous fibrosis. However, collagenous fibrosis was not enhanced by anticancer drugs in AML patients [9]. In contrast, there are reports of marked BMF at APL diagnosis [5–7]. Abou Dalle et al. summarized the characteristics of nine patients in previously published reports, including their own [5]. Among them, four patients showed resolution of BMF after treatment. Mori et al. showed transforming growth factor-*β*_1_ (TGF-*β*_1_) secreted by APL cells promoted BMF [7]. Unlike the above reports, our case presented with irreversible and progressive BMF as treatment progressed (Figure 4). CR was achieved after induction therapy and was maintained until the third consolidation therapy, suggesting that ATRA treatment or APL cells did not cause BMF. In cases of MPN, BMF is associated with abnormal number or function of megakaryocytes and platelets, which secrete cytokines with fibrogenic potential (such as TGF-*β* and platelet-derived growth factor) [4, 10]. In our case, bone marrow biopsies after consolidation chemotherapies showed megakaryocytes were within the normal range without dysplasia. Splenomegaly was not observed in a CT scan just before death. Thus, it was unlikely that MPN developed during cytotoxic chemotherapy administration. *JAK2* or other mutations could not be tested due to lack of preserved specimens. BMF as a side effect of cytarabine or anthracyclines has not been reported in the literature.

In the bone marrow biopsy at the time of diagnosis, the border of bone trabecula in contact with APL cells was osteolytic (Figures 5(a) and 5(b)). Although the patient had coccygeal pain, magnetic resonance imaging of the pelvis during ATRA treatment demonstrated no apparent osteolytic lesions. Among hematological disorders, multiple myeloma and adult T-cell leukemia are the most frequent causes of hypercalcemia associated with bone resorption [11]. In these diseases, osteoclastic differentiation/activity is enhanced through PTH-rP and RANKL signaling pathways [11]. Despite this, hypercalcemia in AML is rare [11]. There were no previous reports of PTH-rP-positive AML cells from patients. Human cell line HL-60 cells, established from patient's APL cells, were able to secrete PTH-rP when stimulated with phorbol 12-myristate, 13-acetate (PMA), but not when unstimulated [12]. In our case, the serum calcium level on patient admission was not tested because she had no signs or symptoms of hypercalcemia. Although APL cells in our case were partially PTH-rP-positive (Figure 5(c)) and RANKL-negative (data not shown) by immunostaining, almost no osteoclasts were observed by TRAP staining (data not shown). Thus, the significance of PTH-rP-positive APL cells in our case was unknown.

Bone marrow biopsy after induction with ATRA showed multilayered osteoblasts along bone trabecula (Figure 6). There were also small pieces of trabeculae, which may have been regenerated. As it was only observed after induction with ATRA but not after two courses of consolidation chemotherapy, ATRA might be the cause of this phenomenon. ATRA acts on osteoblasts and osteoclasts and causes bone remodeling [13]. It would be worthwhile to examine the correlation between ATRA blood levels and osteoblast proliferation *in vivo* with a large sample size.

In our case, the patient had bone pain during the induction and the first two courses of consolidation chemotherapy. In the literature, bone pain was observed in 14 of 101 newly diagnosed APL patients during induction by ATRA with or without chemotherapy [14] and also seen in relapsed/refractory APL patients [15]. Bone pain due to cytarabine has been reported as part of “cytarabine syndrome” which is observed 6 to 12 hours after administration of high-dose cytarabine [16, 17]. There is a report of cytarabine syndrome during continuous injection of low-dose cytarabine (20 mg/m^2^/day), but bone pain was not observed [18]. Primary and secondary myelofibrosis accompanies progressive bone pain in extremities due to extramedullary hematopoiesis [19]. In our case, bone pain occurred on day 18 of induction therapy with ATRA, on days 7–10 of the first consolidation therapy, and days 4–12 of the second consolidation therapy with a relatively small amount of cytarabine by continuous injection. Pain mainly occurred in the coccyx. However, no pain was recorded during the third consolidation therapy. Since the mechanism of bone pain caused by ATRA and cytarabine is unknown, it is unclear whether our patient was idiosyncratic or if bone pain was associated with the progression of BMF.

Phlegmonous gastritis (PG) is a rare but occasionally fatal stomach bacterial infection [20, 21]. Common symptoms include fever, epigastralgia, nausea, and vomiting, which resemble peritonitis and often present as acute abdomen. Hematemesis is also usually observed. Laboratory tests show elevation of white blood cell counts and CRP. Computed tomography demonstrates thickening of the stomach wall (localized or generalized) and the low-density area inside the wall. Gastroendoscopy shows thickened folds and extensive ulceration. The predisposing factors to PG are chronic gastritis, gastric trauma, alcoholism, debilitation, and achlorhydria caused by proton pump inhibitor use, infection, and immunosuppression. Treatment consists of antibiotics and surgical resection. There are only a few reports on PG in immunocompromised patients. In the literature, only four cases of PG were reported in blood disorders (multiple myeloma, gastric lymphoma, chronic myelomonocytic leukemia, and ALL) [21–24]. In our case, the patient suffered from pneumocystis pneumonia during prolonged myelosuppression, suggesting an extremely immunocompromised state. In addition, she was taking a proton pump inhibitor, another predisposing factor to PG. *Enterobacter cloacae*, *Enterococcus* species, and *Staphylococcus* species were detected in the gastric fluid culture, while the blood culture was negative. The clinical course was acute, and she died within two days. Physicians should keep in mind PG as a differential diagnosis of acute abdomen in patients with hematological diseases.

In AML patients, bone marrow biopsy might not be routinely performed for follow-up because minimal residual disease is mainly evaluated by karyotyping, FISH, and qPCR [2]. However, as our case illustrates, bone marrow biopsy can demonstrate progressive BMF, which predicts imminent myelosuppression and a resulting risk of severe infection.

## 4. Conclusion

Here, a rare case of APL with progressive BMF was reported. Evaluation of BMF by bone marrow biopsy after each treatment cycle may serve as a predictor of persistent myelosuppression leading to severe infection. Since there has been no report on PTH-rP-positive AML cells, association regarding stratification of osteoblasts, BMF, and bone pain during treatment for APL, further study is warranted.
